# Gonadal Steroids and Sperm Quality in a Cohort of Relapsing Remitting Multiple Sclerosis: A Case-Control Study

**DOI:** 10.3389/fneur.2020.00756

**Published:** 2020-08-04

**Authors:** Emanuele D'Amico, Aurora Zanghì, Giovanni Burgio, Clara Grazia Chisari, Rosita Angela Condorelli, Sandro La Vignera, Aldo Eugenio Calogero, Francesco Patti

**Affiliations:** ^1^Department “G.F. Ingrassia”, MS Center, University of Catania, Catania, Italy; ^2^Department of Clinical and Experimental Medicine, University of Catania, Catania, Italy

**Keywords:** multiple sclerosis, testosterone, hypothalamic-pituitary-testicular axis, disease modifying therapies, disease activity

## Abstract

**Introduction:** Evaluation of the hypothalamic-pituitary-testicular axis and sperm analyses are not a standard examination of patients with Relapsing-Remitting Multiple Sclerosis (RRMS).

**Methods:** This is a prospective-case-controlled study. Patients, aged 18–55, with a confirmed diagnosis of RRMS, naïve to any DMT were enrolled. Controls were men with normal evaluation who acceded to the Andrology Center of Catania in a contemporary matched randomized fashion to the group of RRMS patients. The aim of the study is to evaluate gonadal steroids and sperm quality in men at the time of RRMS diagnosis and 12 months following the first disease modifying treatment (DMT).

**Results:** Out of 41 patients with RRMS, 38 were included in the study (age 40.3 ± 12.3) to be compared with matched controls. Patients with RRMS showed no differences in gonadal steroids or sperm parameters, except for free testosterone (fT) plasma levels, which were lower in RRMS patients than controls (median 0.09 *vs*. 1.4, *p* < 0.0001). The correlation analyses, corrected for age and Body Mass Index, did not reveal any correlation between hormonal/sperm parameters and level of disability or disease activity at onset. Additionally, 12 months following the start of DMT, there were no differences in gonadal steroids and sperm quality compared to baseline.

**Conclusions:** Results suggest that RRMS may not have an impact on fertility status but prospective long-term studies are needed.

## Introduction

Relapsing-remitting multiple sclerosis (RRMS) is a severe inflammatory degenerative disease of the central nervous system (CNS), with typical onset occurring in the third to fourth decade of life (1). While its etiology is not defined, autoimmunity (influenced by environmental and genetic factors) is still considered a major player in the disease etiopathogenesis (1).

RRMS shows a three times higher disease prevalence in women than men but causes disability faster in men (1). It has been suggested that the gender dimorphism may be linked to the effects of sex hormones on the immune system and parenchymal CNS cells. Indeed, the modifications of sexual hormone milieu in women at puberty, during pregnancy, puerperium, and after menopause may significantly modify the course of RRMS (2).

In men, 98% of plasmatic testosterone (T) is irreversibly converted to the active metabolite 5α-dihydrotestosterone (5α-DHT), which binds the androgen receptor with a higher affinity than T. 5α-DHT is a DNA-binding ligand-activated transcription factor that regulates the expression of the target gene in many cell lines, including leukocytes (3, 4). There are several important effects of T on immune function, such as the shift from the Th1 to Th2 phenotype, leading to an increased production of interleukin-5 (IL-5) and interleukin-10 (IL-10) and decreased pro-inflammatory cytokines such as interferon-(IFL- γ), tumor necrosis factor-α (TNF-α), and interleukin-17 (IL-17), with a consequent impact on lymphocyte proliferation and differentiation. Several studies have described the neuroprotective effect of T, given its ability to cross the blood–brain barrier and directly act on neuronal cells (5, 6).

In patients with MS, a relative androgen deficiency has been suggested and lower levels of T were correlated with disability severity and decreased cognitive function. In one pilot study, 10 men with MS were treated with 10 g of T gel (100 mg) in a cross-over design (6-months of observation followed by 12 months of treatment), demonstrating a significant slowing of brain atrophy measured from monthly magnetic resonance imaging (MRI) and improvement in cognitive testing (7).

Less investigated has been the activity of hypothalamus-pituitary-testicular (HPT) axis and sperm parameters in patients with MS (7). Neuroinflammation can disrupt this axis, with consequences on hormone production and reproductive function; therefore, this topic needs to be more deeply evaluated. Recent public health announcements consider semen quality as an index of general health, further suggesting the need for this to be evaluated in men with MS.

The current case-controlled prospective pilot study was designed to evaluate gonadal steroids and sperm parameters of men at both onset of RRMS and 12 months following their first disease modifying treatment (DMT).

## Methods

### Participants

This is a prospective case-control pilot study, conducted at the tertiary MS Center of Catania, the main referral center in Eastern Sicily (Italy), with a wide catchment area including the regional areas of Catania, Syracuse, Enna, and Ragusa (with more than 2,000,000 inhabitants). Patients with RRMS who were admitted to the MS Center from February 1st to August 31st, 2018 were screened according to the following criteria:

Inclusion Criteria

(a) male gender; (b) age between 18 and 55 years; (c) a definitive diagnosis of RRMS per revised McDonald criteria (8); and (d) have not been exposed to any DMT.

Exclusion Criteria

(a) history of any known disease which can cause endocrine abnormalities (e.g., diabetes, other autoimmunity diseases, etc.) except hypogonadism; and (b) diagnosis of alcohol and/or drug dependence.

Patients were prospectively followed over 12 months and then re-evaluated. Controls were men with normal evaluation screened for gonadal steroids and sperm parameters who acceded to the Andrology Center of Catania in a contemporary matched randomized fashion (for age and Body Mass Index, BMI) to the group of RRMS patients.

### Protocol Approvals Standard, Registrations, and Patient Consents

The local ethics committee (Comitato Etico Catania 1, n. 0011849/2019) at Policlinico Vittorio Emanuele, Catania, Italy approved the study protocol. Subjects with RRMS provided written informed consent. The current study was conducted in accordance with the ethical principles of the Declaration of Helsinki as well as with the appropriate national regulations.

### Data Collection and Definitions

Subjects with RRMS were evaluated for neurological and andrological status both at enrollment and 12 months following the start of the prescribed DMTs.

### Neurological Assessment

The following data were collected from each subject:

- *Demographic data*. Data collected included date of birth, BMI, smoking status, and number of children the subject had fathered.
- *Clinical data*. Included the date of MS onset, type of MS, and level of disability as assessed by the Expanded Disability Status Scale (EDSS). Symptoms at onset were classified as involving supratentorial, visual pathway, cerebellar/brainstem, and spinal cord symptoms.
- *Radiological data*. The scanning sessions of the brain and spinal MRI sequences were T1- and T2-weighted sequences acquired only with 1.5 Tesla scanners and longitudinally with the same scanner. Spinal cord MRI included both cervical and thoracic tracts.
  The T1-weighted sequences were acquired before and after intravenous injection of gadolinium contrast agent (0.1 mmol/kg).
  A cerebral and spinal cord MRI acquired within 3 months before or 1 week after the treatment start was considered as a baseline MRI.
  The numbers of brain and spinal (cervical and thoracic) MRI lesions on T2-weighted and post-contrast T1-weighted sequences were recorded at baseline and after 12-month of follow-up from the beginning of DMT.
  Lesions and imaging were compared with the support of available software (Carestream®Healthcare Information Systems software).

- *Cognitive impairment as evaluated by the Brief Repeatable Battery Neuropsychology (BRB-N) and Stroop test*. BRB-N evaluation included Selective Reminding Test Long-Term Storage, Selective Reminding Test Long-Term Retrieval, Selective Reminding Test Delayed Recall, 10/36 Spatial Recall Test, 10/36 Spatial Recall Test Delayed, Symbol Digit Modalities Test (SDMT), Paced Auditory Serial Addition Task 3 (PASAT 3), and Word List Generation (WLG) (9, 10). Also, Stroop Test was performed. Patients were classified as having cognitive impairment when they fail at least three neuropsychological tests belonging to the BRB (11) and Stroop test, in the light of previous studies using the same neuropsychological battery and reporting that less than 5% of healthy controls failed more than three tests (10, 12, 13).
- *First prescribed DMT*. Included the type of DMT, start date and any withdrawn or switch date.
  All neurological evaluations were performed by neurologists (ED, AZ, FP) trained in neurostatus examinations (https://www.neurostatus.net). Data were retrieved from a computerized database, iMed^©^ software (iMed^©^, Merck Serono SA; Geneva) in accordance with the Italian MS Web registry guidelines.

### Andrological Assessment

The andrological assessment was conducted in the Division of Andrology and Endocrinology at the University of Catania. Each patient underwent the following blood testing and laboratory/instrumental tests:

- *Hormonal Measurements*. Blood sampling was performed at 8.00–8.30 am, after at least 8 h of sleep. The serum concentrations of luteinizing hormone (LH), Follicle-stimulating hormone (FSH), and total testosterone (TT) were measured by electrochemiluminescence using a Hitachi-Roche equipment (Cobas 6000, Roche Diagnostics, Indianapolis, IN, USA). Normal ranges were defined as LH = 1.6–9.0 mIU/mL^−1^, FSH = 2.0–12.0 mIU/mL^−1^, TT = 3.5–8.0 ng/mL^−1^ (the gray-zone for TT was defined 2.3–3.4 ng/mL^−1^). The blood tests also included a complete blood count and differential, platelet count, sex hormone binding globulin (SHBG), albumin, aspartate, and alanine aminotransferase, alkaline phosphatase, total bilirubin, creatinine, and electrolytes. Free T (fT) was calculated using the serum values of TT, SHBG and albumin.
- *Sperm analysis*. Seminal fluid samples were collected by masturbation in a sterile container following 2–7 days of sexual abstinence and were transported to the laboratory within 30 min from ejaculation. Sperm analysis was performed according to the 2010 WHO criteria (Appendix 1).

Hypogonadism was defined using the latest definition proposed by the Endocrine Society guidelines (14).

## Outcomes

The main objectives of the study were to identify any correlation between disease characteristics at onset and hormonal/sperm parameters and investigate if hormonal/sperm parameters were associated with longitudinal changes of clinical/radiological parameters 12 months following the first prescribed DMT.

### Statistical Analysis and Covariate Definitions

Baseline characteristics and gonadal steroids are reported as frequencies (%), mean with standard deviation, or median with interquartile range (min-max). Sperm parameters were calculated assuming a binomial distribution with 95% confidence intervals. Proportions among categorical variables were compared using a χ^2^ analysis.

To examine the associations between androgen measures and other hormones, Pearson correlation coefficients and partial Pearson correlation coefficients adjusting for age and BMI were calculated. The primary cross-sectional analysis was the correlation between total testosterone, free T, and EDSS at the first visit date. Secondarily, the cross-sectional correlation between total testosterone and number of relapses in the year prior to diagnosis and number of brain MRI lesions on T2 and T1 gadolinium weighted sequences were investigated. Exploratory analyses were run for the other androgen variables (free testosterone, SHBG, and sperm parameters) using similar models. A 2-tailed *p*-value of lower than 0.05 was considered significant.

The longitudinal analyses examined the association between baseline testosterone levels and longitudinal changes in clinical and radiological parameters using generalized estimating equations.

According to the AIC criterium, we selected the model with the best statistical inferential properties. All the models were estimated using the Breslow's tie correction.

For each outcome and predictor pair, the mean change in each outcome was assumed to be linear with time and the primary analysis was the effect of the baseline testosterone measurement on the change over time. Each model was fit assuming an identity link function and an exchangeable working correlation structure. Robust standard errors were used to calculate *p*-values (15). Each model was corrected for: age (as continuous variable), BMI (as continuous variable), time from first symptom to diagnosis (disease duration, in months, continuous variable) and line of DMT prescribed (as categorical variable).

These models were conducted for the following outcomes: EDSS (at baseline and after 12 months from enrolment), number of brain MRI lesions on T2 weighted sequences (at baseline and after 12 months from enrolment), number of brain MRI lesions on T1-gadolinium weighted sequences (at baseline and after 12 months from enrolment), and for number of relapses (within 12 months before diagnosis and after 12 months from enrolment). They were built using the proportional odds link and conclusions were the same as those reported below.

The formula employed for sample size calculation was sample size value for finite population. Giving annual incidence of RRMS in Catania and total population in province of Catania (ISTAT), setting a pre-study standard deviation to 50% because the behavior of the population under study is not known (pilot-study), with a Z-score of 1.96 and margin of error (e) of 0.05 and considering 6 months of enrolment, the population of the study will have to reach 26.

SPSS version 21.0 was used for all analyses (IBM SPSS Statistics 21, IBM^©^, Armonk, NY, USA).

## Data Availability Statement

Anonymized data will be shared upon request from any qualified investigator for the sole purpose of replicating procedures and results presented in the current report, provided that the data transfer is in agreement with EU legislation on the general data protection regulation.

## Results

**Figure 1:**
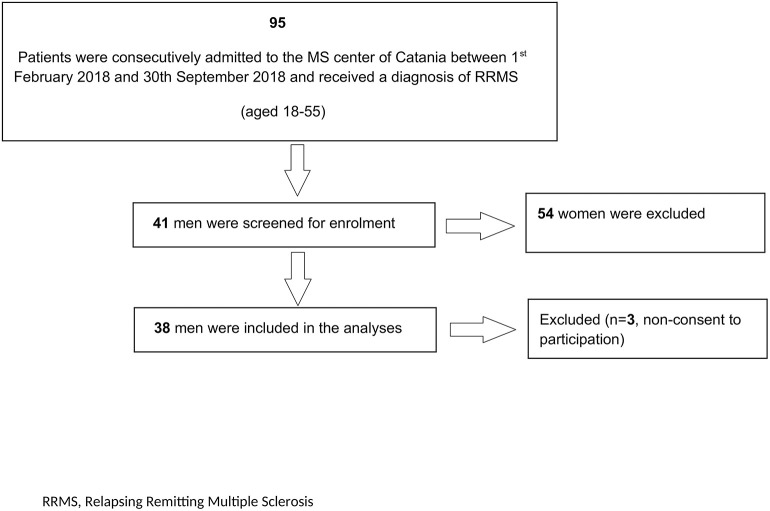
Patients' selection flow chart. RRMS, relapsing remitting multiple sclerosis.

Out of 41 screened patients, 38 were enrolled in the current study with three excluded due to non-consent of participation (Figure 1). The mean age of the 38 patients enrolled was 40.3 ± 12.3 years old and the median EDSS score was 1.5 (IQR 1.0–2.0). Fifteen patients were childless and out of them, twelve did not desire offspring at the time of enrolment. Table 1 summarizes other relevant demographic and clinical characteristics.

**Table 1:** Demographic and clinical characteristics at enrolment.

Variables^*^	
Age	40.3 ± 12.3
Smokers, n (%)	22 (57.9)
BMI	23.7 ± 2.1
Men childless	15 (39.5)
EDSS (median, IQR)	1.5 (1–2.0)
Time from first symptoms (disease duration, months)	11.8 ± 6.4
N. of relapse 2 years before diagnosis	1.4 ± 1.3
N. of relapse 1 year before diagnosis	1.3 ± 0.6
Type of onset, n (%)	
*Visual*	2 (5.4)
*Spinal*	6 (16.2)
*Pyramidal*	0
*Cerebellar*	0
*Multidomain*	29 (78.4)
N. brain MRI lesions on T2-weighted sequences	16 ± 11.2
N. spinal cord MRI lesions on T2-weighted sequences	3.0 ± 2.3
N. brain MRI lesions on T1-gadolinium weighted sequences	0.4 ± 0.9
N. spinal cord MRI lesions on T1-gadolinium weighted sequences	0.5 ± 0.8
Patients with cognitive impairment at enrollment, n (%)	7 (5.4)
Number of impaired tests at BRB	0.7 ± 0.8

*BMI, body mass index; BRB, brief repeatble battery; EDSS, expanded disability status scale; IQR, interquartile range; MRI, magnetic resonance imaging; N, number*.

* *Data are expressed as mean ± standard deviation when otherwise specified*.

### Hormonal Assessment and Sperm Parameters

The median values of TT and calculated free-T are shown in Table 2. Among the RRMS patients, 3 (7.9%) patients had hypogonadism with no compensatory elevation of LH and 5 (13.2%) patients were in the gray zone. Patients with RRMS showed no differences in fT values, while fT was higher in healthy controls (*p* < 0.001). Among controls, hypogonadism was present in three patients (7.9%) and eight were in the gray zone (21.1%). Patients with RRMS, compared to healthy controls (according to WHO 2010 reference values), did not show any differences in terms of seminal fluid volume, total sperm count, or total and progressive motility (Table 2).

**Table 2:** Hormonal assessment and spermatic parameters in RRMS patients at enrolment and in an age-BMI matched control population.

Variables^*^	**Cases**	**Controls**	***p*-value**
**Hormonal parameters**			
TT	4.8 (3.4–6.2)	5.3 (4.4–6.6)	ns
**freeT**	**0.09 (0.07–0.11)**	**1.4 (0.9–1.91)**	0.000^**^
FSH	3.8 (2.4–5.9)	3.6 (2.3–4.1)	ns
LH	3.3 (2.3–4.5)	3.1 (2.3–4.1)	ns
SHBG	32.4 (27.4–43.4)	32.5 (26.1–41.7)	ns
**Spermatic parameters**			
Total number	179.3 ± 165.7	196.8 ± 129.6	ns
Concentration	59.4 ± 44.6	74.5 ± 37.6	ns
Volume	3.2 ± 1.7	2.6 ± 1.2	ns
Morphology	6.3 ± 3.5	7.2 ± 4.4	ns
Progressive motility	18.0 ± 10.6	19.4 ± 12.6	ns

* *Data are expressed as mean ± standard deviation and as median (IQR)*.

** *significant p-values are in bold*.

*BMI, body mass index; IQR, interquartile range; FSH, follicular stimulant hormone; LH, luteinizing hormone; N, number; SHBG, sex hormone binding globulin; TT, total testosterone; freeT, free testosterone*.

### DMT's Prescription

**Figure 2:**
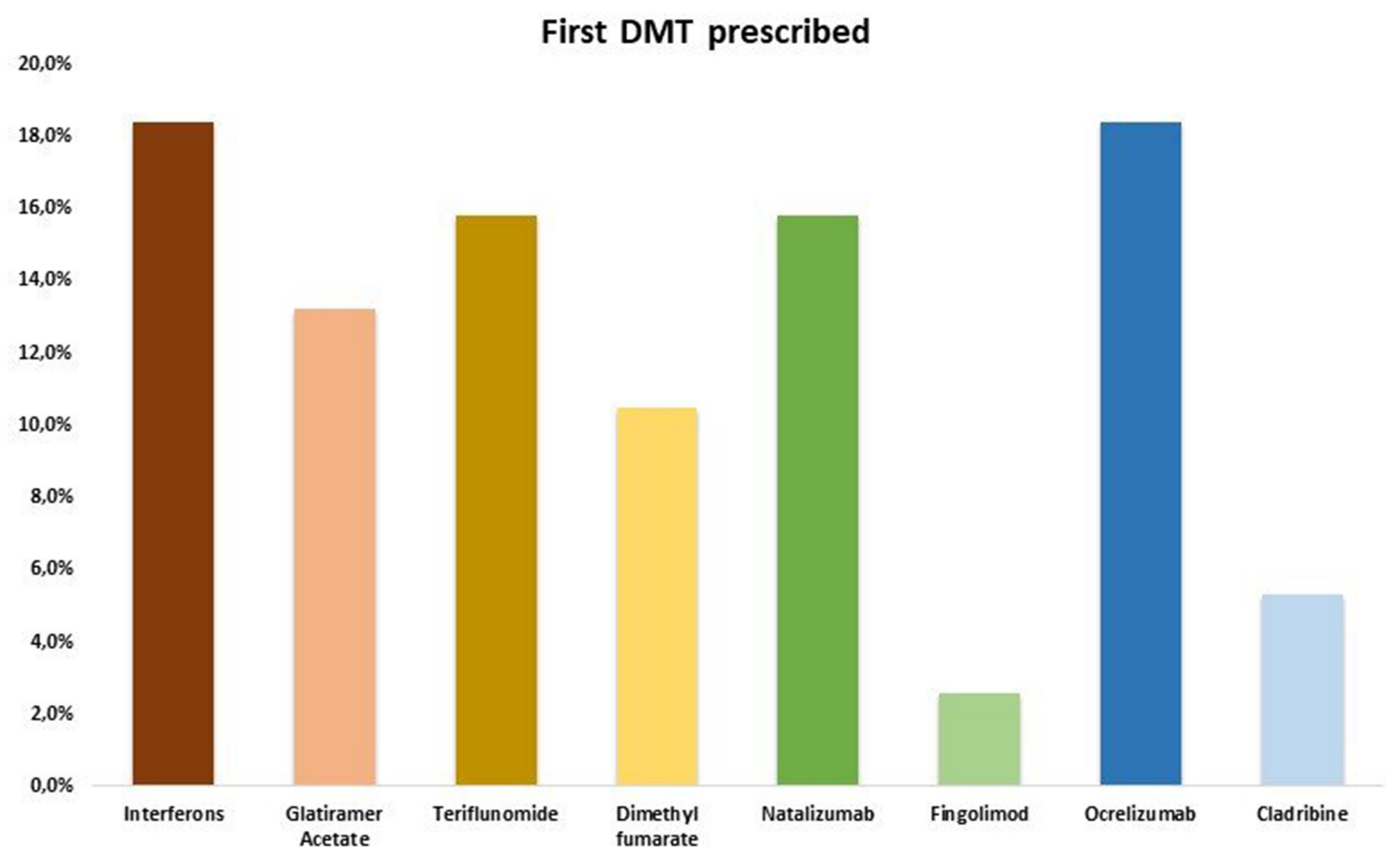
First DMT prescribed. DMT, disease modifying therapy.

All RRMS patients started a DMT within 3 months from onset and none discontinued treatment prior to the last follow-up (Figure 2). No differences were found about the rate of first and second line DMTs prescribed: 20/38 (52.6%) RRMS patients were on first line DMT, whilst 18/38 (47.4%) were on second line DMT.

### Cross-Sectional Analyses

The correlation analyses between gonadal steroids/sperm parameters and disease characteristics at onset (see methods) showed no primary correlation alone nor when corrected for age and BMI (see Tables 3A,B).

**Table 3A:** Association between baseline androgens and baseline clinical (EDSS. number of relapses 12 months before diagnosis), baseline radiological (N. MRI T2 and T1-gadolium weighted lesions) and n. of impaired tests cross-sectional outcomes.

**Cross-sectional**					
		**Unadjusted**		**Age and BMI adjusted**	
	**Hormone**	**R**	***p***	**R**	***p***
EDSS	Testosterone	0.094	0.694	0.133	0.612
	freeT	0.042	0.859	0.087	0.739
N. brain MRI T2-weighted lesions	Testosterone	−0.208	0.250	−0.184	0.013
	freeT	−0.174	0.333	0.480	0.961
N. brain MRI T1-gadolinium weighted lesions	Testosterone	0.021	0.909	−0.080	0.762
	freeT	−0.040	0.824	0.005	0.986
N. of relapses 12 months before diagnosis	Testosterone	−0.141	0.441	−0.155	0.553
	freeT	−0.103	0.573	−0.103	0.693
N. of cognitive tests impaired	Testosterone	−0.345	0.091	−0.280	−0.243
	freeT	−0.344	0.092	0.195	0.264

*EDSS, expanded disability status scale; Gad, gadolinium; N, number; MRI, magnetic resonance imaging*.

*For the EDSS correlations, Spearman's correlation coefficients are reported; for the others, Pearson's correlation coefficients are reported*.

**Table 3B:** Association between baseline sperm parameters and baseline clinical (EDSS. number of relapses 12 months before diagnosis), baseline radiological (N. MRI T2 and T1-gadolium weighted lesions) and cognitive (number of impaired tests) cross-sectional outcomes.

**Cross-sectional**					
		**Unadjusted**		**Age and BMI adjusted**	
	**Sperm parameter**	**R**	***p***	**R**	***P***
EDSS	Volume (ml)	−0.093	0.862	0.054	0.815
	Count (10^6^/ml)	0.165	0.474	0.000	1
	Total count (10^6^)	0.035	0.880	0.225	0.326
	Motility (%)	−0.292	0.199	−0.275	0.227
	Progressive (%)	0.020	0.933	0.035	0.882
	Morphology (%)	0.231	0.314	0.157	0.496
N. brain MRI T2-weighted lesions	Volume (ml)	−0.260	0.223	−0.230	0.323
	Count (10^6^/ml)	0.132	0.457	−0.411	0.136
	Total count (10^6^)	0.132	0.458	−0.235	0.271
	Motility (%)	0.132	0.457	0.008	0.491
	Progressive (%)	0.022	0.901	0.210	0.293
	Morphology (%)	0.214	0.225	0.412	0.135
N. brain MRI T1-gadolinium weighted lesions	Volume (ml)	0.103	0.561	−0.203	0.200
	Count (10^6^/ml)	0.103	0.561	0.396	0.146
	Total count (10^6^)	0.157	0.374	−0.235	0.271
	Motility (%)	0.224	0.203	0.008	0.491
	Progressive (%)	0.097	0.587	0.210	0.293
	Morphology (%)	0.047	0.792	0.412	0.135
N. of relapses 12 months before diagnosis	Volume (ml)	0.127	0.482	0.049	0.450
	Count (10^6^/ml)	0.162	0.366	−0.186	0.316
	Total count (10^6^)	−0.125	0.714	−0.329	0.194
	Motility (%)	0.035	0.845	−0.092	0.407
	Progressive (%)	−0.147	0.414	0.126	0.373
	Morphology (%)	0.155	0.390	−0.541	0.066
N. of cognitive tests impaired	Volume (ml)	−0.058	0.881	0.485	0.135
	Count (10^6^/ml)	−0.236	0.218	0.035	0.470
	Total count (10^6^)	−0.086	0.656	0.556	0.097
	Motility (%)	−0.149	0.442	0.406	0.183
	Progressive (%)	−0.253	0.185	0.437	0.163
	Morphology (%)	−0.105	0.588	0.613	0.072

*EDSS, expanded disability status scale; Gad, gadolinium; N, number; MRI, magnetic resonance imaging*.

*For the EDSS correlations. Spearman's correlation coefficients are reported*.

### Longitudinal Analyses-12-Months Follow-Up

Following 12 months, no differences were found in either gonadal steroids or sperm parameters in the current cohort (Table 4). Additionally, our analyses assessing the relationship between baseline TT and longitudinal clinical changes (EDSS, MRI lesions load, and relapses) demonstrated no associations (Table 5).

**Table 4:** Hormonal assessment and spermatic parameters in RRMS patients at enrolment and after 12 months.

Variables^*^	**AT Enrolment**	**After 12 months**	***p*-value**
**Hormonal parameters**			
TT	4.8 (3.4–6.2)	5.1 (3.9–6.7)	ns
freeT	0.09 (0.07–0.11)	0.09 (0.07–0.11)	
FSH	3.8 (2.4–5.9)	2.9 (2.1–4.1)	ns
LH	3.3 (2.45)	2.7 (1.7–3.2)	ns
SHBG	32.4 (27.4–43.4)	36.4 (30.4–48.9)	ns
**Spermatic parameters**			
Total number	179.3 ± 165.7	129 ± 127.4	ns
Concentration(10^6^/ml)	59.4 ± 44.6	50 ± 37	ns
Volume (ml)	3.2 ± 1.7	3 ± 1.7	ns
Morphology (%)	6.3 ± 3.5	6.5 ± 3.6	ns
Total motility (%)	70 ± 49.5	70 ± 51.1	ns
Progressive motility (%)	18.0 ± 10.6	20.7 ± 10.3	ns

* *Data are expressed as mean ± standard deviation and as median (IQR)*.

*IQR, interquartile range; FSH, follicular stimulant hormone; LH, luteinizing hormone; N, number; SHBG, sex hormone binding globulin, TT, total testosterone; freeT, free testosterone*.

**Table 5:** Association between androgen levels and longitudinal changes in clinical and radiological outcomes.

**Longitudinal changes**					
	**Hormone**	***Estimate***	**95% CI**		***p-value***
			**Lower**	**Upper**	
EDSS	Testosterone	0.035	−0.128	−0.199	0.673
	FreeT	0.240	−2.25	2.73	0.850
Relapses	Testosterone	0.206	−0.494	0.906	0.564
	FreeT	3.76	−21.88	29.41	0.774
Lesions load at MRI T2-weighted sequences	Testosterone	−0.144	−3.16	0.271	0.099
	FreeT	−13.7	−44.27	16.7	0.376
Lesions load at MRI T1-gadolinium weighted sequences	Testosterone	0.197	−0.320	0.807	0.498
	FreeT	3.12	−19.76	18.96	0.633

*GEE analysis for the yearly changes in EDSS, MRI lesions load and relapses for 100 unit of increase of baseline TT*.

*freeT, free testosterone; GEE, generalized estimation equation; MRI, magnetic resonance imaging; TT, total testosterone*.

## Discussion

In the current cohort, patients with RRMS showed a prevalence of hypogonadism comparable to data reported in population-based studies, in which the prevalence of male hypogonadism is described from 2.1 to 12.8% (16). Furthermore, sperm parameters did not differ from controls. There was no change in gonadal steroids or sperm parameters after 12 months from the first prescribed DMT nor were clinical and radiological variables at last follow-up associated with baseline gonadal steroids and sperm parameters.

Very limited data are available on this topic and, to date, no prospective studies have been performed.

The role of T in influencing MS onset and course is matter of debate. In studies on autoimmune diseases such as systemic lupus erythematosus or rheumatoid arthritis, it has been suggested that low testosterone levels may be present at disease onset.

In a recent observational study on 96 men with RRMS (mean age of 40 years, EDSS of 1.1, and disease duration of 4.6 years), that 39% were hypogonadal (total testosterone <288 ng/dl), demonstrating an association of low baseline TT levels and disease severity (11, 17). The frequency of MS in men is three times less than in women, however, they are more prone to develop severe disability, recover poorly from relapses, and experience increased rates of brain atrophy (18). Unfortunately, limited data currently exists on the effects of T supplementation in men with MS. In a cross-over pilot study, 10 patients with RRMS (aged <65 years) received a daily treatment with 100 mg T gel for 12 months (19). After T treatment, there was a significant improvement in the cognitive performance (*p* = 0.008) as measured by the Paced Auditory Serial Addition Task and a slowing of atrophy, as measured with MRI (*p* < 0.001). No significant effect of T was found on T1 gadolinium lesion numbers or whole brain volume, however (20). These data may suggest an immunomodulatory and a potentially neuroprotective effect of T treatment in MS, however, additional large, multicenter studies are needed to confirm these results.

We did not find any differences of sperm quality compared to controls, nor were any associations found with clinical/radiological characteristics at onset. In a cross-sectional study in which a total of 68 patients with MS (mean age 44.2 years, range 31–56 years) were compared to 48 healthy volunteers, Safarinejad et al. (7) described abnormal sperm parameters in 14 patients with RRMS (out of 66). In another study, 73 patients with MS represented an ancillary subpopulation of 4,480 patients (with cancers and chronic inflammatory diseases) (21). Of these patients, 27.4% had sperm abnormalities (compared to WHO 2010 reference values) (21). Given these findings, it is not currently clear if systemic inflammation in MS impacts testicular physiology and sperm production (22).

The MS therapeutic landscape has impressively changed in the last few years, with very striking and effective drugs being released. The increase in efficacy, however, has been accompanied by an increase in safety concerns. In this regard, no attention has been paid on men's fertility status in either research or clinical practice. In the pursuit of a personalized treatment, future studies are needed to clarify the impact of new DMTs on spermatogenesis and male fertility (23, 24).

In summary, the current study finds no specific issues in general male fertility status and that neither gonadal steroids nor sperm quality was associated with disability accrual after 12 months on DMTs, but this short follow up cannot allow definitive conclusions.

## Data Availability Statement

The datasets generated for this study are available on request to the corresponding author.

## Ethics Statement

The local ethics committee (Comitato Etico Catania 1, no. 0011849/2019) at Policlinico Vittorio Emanuele, Catania, Italy approved the study protocol. Subjects with RRMS provided written informed consent. The current study was conducted in accordance with the ethical principles of the Declaration of Helsinki as well as with the appropriate national regulations.

## Author Contributions

ED'A: study concept and design, acquisition of data, critical revision of manuscript for intellectual content. AZ: study concept and design, statistical analysis. GB: acquisition of data and drafting manuscript. CC, RC, and SL: acquisition of data. AC and FP: study concept and design, supervision. All authors contributed to the article and approved the submitted version.

## Conflict of Interest

The authors declare that the research was conducted in the absence of any commercial or financial relationships that could be construed as a potential conflict of interest.
