# Supplementary material for: Gonadal Steroids and Sperm Quality in a Cohort of Relapsing Remitting Multiple Sclerosis: A Case-Control Study
Source: Front Neurol. 2020 Aug 4;11:756. doi: 10.3389/fneur.2020.00756 (PMC7417674; doi:10.3389/fneur.2020.00756)
Supplement: Supplementary file 1 [file Table_1.DOCX]

**Appendix 1. WHO reference values 2010**

| Volume (ml) | ≥1.5 |
| --- | --- |
| Count (10^6^/ml) | ≥15 |
| Total count (10^6^) | ≥39 |
| Motility (%) | ≥40 |
| Progressive (%) | ≥32% |
| Vitality (%) | ≥58 |
| Morphology (%) | ≥4 |
| Leukocytes (10^6^/ml) | 1.0 |

***Esteves et al. Urology, 2012.**
